# Antibacterial activity of peptide derivatives of dermaseptins against multidrug-resistant *Klebsiella pneumoniae* and Staphylococcus epidermis

**DOI:** 10.1016/j.bbrep.2026.102449

**Published:** 2026-01-29

**Authors:** Houda Haddad, Reyadh R. AL-Rashidi, Ahmed Loghmari, Wissal Sahtout, Raja Boukadida, Rihem Dahmene, Emeny Ettouil, Houcemeddine Othman, Ines Ouahchi, Amira Zaϊri

**Affiliations:** a BIOLIVAL Laboratory, the Higher Institute of Biotechnology of Monastir ISBM, University of Monastir, Monastir, 5000, Tunisia; b Biochemistry department, Faculty of Medicine, University of Sousse, Sousse, 4002, Tunisia; c Department of Dentistry, Kut University College, Wasit, Kut, 52001, Iraq; d Urology Department, Sahloul Hospital, Sousse, 4054, Tunisia; e Researsh laboratory "Epidémiologie des maladies mentales, Dépistage et Prise en Charge précoce" LR12ES04, Faculty of Medicine, University of Sousse, Sousse, 4002, Tunisia; f Nephrology Department, Sahloul University Teaching Hospital, University of Sousse, Sousse, 4054, Tunisia; g Research Laboratory LR12SP11, Biochemistry Department, Sahloul University Hospital, Sousse, 4054, Tunisia; h Research Laboratory Heart Failure LR12SP09, Tunisia; i Nephrology Department, Ibn El Jazzar University Hospital, University of Sousse, Kairouan, Tunisia; j Laboratory of Cytogenetics and Reproductive Biology, CHU Farhat Hached, Sousse, 4000, Tunisia; k Biodiversity Cytogenetics, Molecular Genetics and Reproductive Biology, Farhat Hached University Hospital, Sousse, 4000, Tunisia

**Keywords:** Dermaseptin B2, Dermaseptin S4, Derivatives, *Staphylococcus epidermidis*, *Klebsiella pneumoniae*, Infections, Antibacterial activity

## Abstract

The emergence of infections caused by multi-drug resistant bacteria (MDR) to antibiotic treatments poses a significant challenge in the healthcare field. Indeed, the resistance of MDR such as: *Klebsiella pneumonia* and *Staphyloccus epidermidis* to antibiotics has become an increasingly concerning issue, especially in hospital settings, necessitating the development of new therapies and more potent antimicrobial agents. Although numerous conventional antibiotic agents have been developed in recent years, but many of them still present toxicity to eukaryotic cells, despite their significant efficacy against multi-resistant microorganisms. Therefore, antimicrobial peptides (AMPs), particularly, dermaseptins (DRSs), are considered promising candidates against MDR, mainly due to their low toxicity and their different mode of action compared to conventional antibiotics. Indeed, these peptides are generally less likely to lead to the resistance phenomena observed for traditional antibiotics. The objectives of this study were to examine the physicochemical and structural properties of peptide derivatives of dermaseptin S4 and B2, and to ascertain their antibacterial potency against *Staphylococcus epidermis* and *Klebsiella pneumoniae.* The dermaseptin peptide derivatives used in this study were K_4_K_20_S4, K_4_S4(1–16), B2 and K_3_K_4_B2. In this research, we describe the synthesis and the bioactivity of DRSs and their derivatives against *Staphylococcus epidermis* and *Klebsiella pneumoniae*. The cytotoxicity of these compounds was investigated on the HEp-2 cell line by MTT cell viability assay. The cytotoxicity of DRSs was concentration-dependent at microgram concentrations. It was observed that all tested analogs exhibited antibacterial activity with Minimum Inhibitory Concentrations (MICs) ranging from 6.25 to 25 μg/ml and Minimum Bactericidal Concentrations (MBCs) ranging from 12.5 to 50 μg/ml. In summary, these findings indicate that dermaseptins could serve as promising lead compounds for the development of potent antibacterial agents targeting infections caused by *Klebsiella pneumoniae* and *Staphylococcus epidermidis*.

## Highlights

• S4 are more active than B2: at low doses in μg/ml against both bacteria strains.
• All analogs antibacterial activity with (MICs 6.25 to 25 μg/mL ; MBCs 12.5 to 50 μg/mL).
• The cytotoxicity of DRSs was concentration-dependent at microgram concentrations.
• Increasing positive charges via mono- or bi-substitution improves biological activity and reduces cytotoxicity.

## 1 Introduction

The identification of naturally occurring compounds with antibacterial characteristics and the subsequent creation of novel medications based on these compounds constituted a major achievement in medicine throughout the 20th century. This development significantly lowered the death rates from infectious illnesses [1]. Multidrug-resistant microorganisms (MDR) and antibiotic-resistant infections have emerged as a result of the widespread use of antibiotics in healthcare and their misuse. Given that some microbes have genes or mutations that make some antibiotics ineffective, the issue is becoming more and more alarming. The incapacity of current antibiotics to treat antibiotic-resistant bacterial illnesses directly resulted in the deaths of about 1.2 million individuals worldwide in 2019—and probably many more [1]. Gram-negative and gram-positive bacteria developing multidrug resistance (MDR) is a serious worldwide concern. Our research is particularly concentrated on the pathogens: *Klebsiella pneumoniae* and *Staphylococcus epidermidis*.

Among the most dangerous microorganisms for modern medicine is *K. pneumoniae*. It is an extremely resistant member of the aquatic and terrestrial environments of the *Enterobacteriaceae* family. Along with the intestinal microbiome, it also often maintains the mucosal surfaces of humans and animals [2,3]. *K. pneumoniae* is an opportunistic pathogen that may cause infections in the community as well as in hospitals. *K. pneumoniae* has emerged as one of the most frequent etiologic factors in the context of recurrent urinary tract infections due to the emergence of drug-resistant strains of the bacteria [4]. Thus, the management of complex urinary tract infections, such as pyelonephritis and urosepsis, may need the discovery of new antibiotics. Treatment of these infections is often complicated by resistance to various antibiotic classes, such as beta-lactams, quinolones, and aminoglycosides, rather than by the emergence of pathogenicity factors [[5], [6], [7]]. Consequently, infections brought on by *K. pneumoniae* frequently signify an adverse prognosis, with death rates as high as 50 % [8].

We also focus on the pathogen *S. epidermidis* in order to broaden our attention beyond *K. pneumoniae*. *S. epidermidis* is a Gram-positive bacteria as opposed to the Gram-negative *K. pneumoniae*. Natural human microbiota members include *S. epidermidis*, which is consistently found on skin and mucous membranes. By attaching itself to host tissue surface receptors with certain adhesins, it can establish a long-lasting commensal relationship with humans. This exchange may begin at a young age [9]. Due to the bacteria's default standing as commensal, even while commensal *S. epidermidis* isolates show high rates of resistance to clinically important antibiotics, this occurrence is mostly irrelevant for healthy human hosts [9,10]. However, since implanted medical devices like fracture fixation devices and prosthetic joints have become common, *S. epidermidis* has emerged as a major opportunistic pathogen [10,11]. Treatment of *S. epidermidis* infections is significantly hampered by this species' high endemic resistance to antibiotics, even if the majority of *S. epidermidis* strains are still sensitive to more modern medications like tigecycline, daptomycin, linezolid, and dalbavancin [12]. Methicillin resistance in *S. epidermidis* (MRSE) is an important characteristic of infecting isolates since it is often linked to other mechanisms of antibiotic resistance. Resistance to other antibiotics is also commonly observed, especially in MRSE [13]. These drugs include aminoglycosides (encoded in the aacA/aphD gene), trimethoprim-sulfamethoxazole, ciprofloxacin, clindamycin, and erythromycin (encoded by the erm genes). Remarkably, *S. epidermidis* accounts for around 13 % of prosthetic valve endocarditis (PVE) infections, making it the second most common cause after *S. aureus*. A substantial risk of intracardiac abscesses (38 %) and a death rate of 24 % are attributed to these infections [14]. Among coagulase-negative *staphylococci* responsible for urinary tract infections, especially those related with healthcare, *S. epidermidis* is also the most common species [10].

Thus, it is important to have effective strategies to prevent infections with *K. pneumoniae* and *S. epidermidis*. Antibiotics are useless against bacteria because they use a range of approaches. Some of these strategies include the synthesis of enzymes that render antibiotics inactive, lowering the concentration of antibiotics within cells, or even changing the targets of antibiotics [1]. Discovering novel "hot spots" for bacterial metabolism and chemicals which function effectively in them is one way to circumvent multidrug resistance. As a result, we focused on cationic antimicrobial peptides (AMPs) in the current investigation, specifically dermaseptins (DRSs), which have the ability to prevent healthcare-associated infections (HAI), especially those caused by hypervirulent strains. AMPs are small peptides with five to one hundred amino acid residues that demonstrate a wide range of molecular weights (<10 kDa) [15,16]. The innate immune systems of a wide range of species, including mammals, insects, plants, and amphibians, generate these peptides. They come from a variety of sources such as the reproductive system, adipose tissue, hemoglobin, macrophages, neutrophils, and epithelial cells [17].

Among these is the family of AMPs known as dermaseptins (DRSs), which was found in the skin of the Phyllomedusa frog located in South America. Amphibians produce bacterial defense peptides (DRSs), which are mainly composed of 28–34 amino acids and differ greatly among different peptides. Nonetheless, they usually depend on an amphibathic α-helices structure in apolar solvents [18]. The lengths and peptide sequences of DRSs vary significantly. Despite this, they have a number of structural similarities, such as a conserved Trp residue at position 3, a conserved sequence of AA(G)KAALG(N)A in the middle region, and a net positive charge [19]. DRS-S peptides that were isolated from the secretions of *Phyllomedusa sauvagii* haven't been thoroughly investigated in a range of human antibacterial purposes. The potential for these applications to rapidly eliminate diseases and successfully stop germs from growing resistant makes them extremely promising [20]. So far, thirteen DRSs (DS-1 through 13) have all been found and characterized [21].

Drawing on the extant literature on dermaseptins' antibacterial activity, two distinct categories of mechanisms may be discerned: The process that was initially proposed is known as "barrel-stave" and is controlled by DRSs adhering to phospholipids in membranes. This interaction changes the osmotic equilibrium of the cell, which results in membrane permeabilization. After that, transmembrane gaps or channels form, which ultimately result in membrane rupture. The second process, also called the "carpet-like" mechanism, involves positively charged lytic peptides adhering to the negatively charged surface. Complete surface covering is achieved by this mechanism of disintegration, which permits the membrane to breakdown and get impregnated [22].

Several DRSs show a phenomenal ability to stop microbial cells rapidly, efficiently, and permanently without harming mammalian cells [20]. DRSs have a broad spectrum of activity [21] and do not display hemolysis, despite a rare exception of DRS-S4, which has a severe hemolytic impact [23]. Because DRS-B2, which is produced from *Phyllomedusa bicolor* [24], may alter the attachment of agonists of the adenosine A1 receptor, it is often referred to as adenoregulin in this context [25]. DRS-B2 stands out within the DRS family of peptides due to its greater activity, which has prompted extensive study on it. This 33-amino acid, amphipathic (+3) cationic polypeptide has a molecular weight of about 3180 Da, a tryptophan residue at position 3, and six lysine residues [26]. This α-helical peptide can harm the membranes of a variety of microorganisms, particularly bacteria, yeast, fungi, and protozoa. However, the precise mechanism of action is still not fully understood [27].

Therefore, it is important to have effective strategies to prevent infections with *K. pneumoniae* and *S. epidermidis*. These two pathogens were selected as clinically relevant models because they represent distinct bacterial types, Gram-negative and Gram-positive, respectively, both frequently associated with healthcare-associated infections and high levels of multidrug resistance. Their clinical significance and contrasting cell envelope structures make them **ideal models** for evaluating the efficacy and selectivity of newly developed cationic antimicrobial peptides.

To the best of our knowledge, this experiment is the first investigation assessing the antibacterial activity of newly designed dermaseptin B2-derived peptides, including the novel K3K4B2, against *Staphylococcus epidermidis* and *Klebsiella pneumoniae*. These rationally engineered derivatives, developed to modulate charge and hydrophobicity of the native B2 and S4 peptides, have never been previously synthesized or evaluated against these multidrug-resistant clinical isolates.

Consequently, the present study was designed to test the hypothesis that structural modifications of dermaseptin B2 could enhance its antimicrobial potency and selectivity while minimizing cytotoxicity. We aimed to create new peptide derivatives that work better against *K. pneumoniae* and *S. epidermidis*, two clinically important MDR pathogens, by increasing the net positive charge and lowering hydrophobicity. This work thus defines the antibacterial and cytotoxic profiles of these newly synthesized peptides and offers information about structure–activity relationships that may guide the rational design of next-generation antimicrobial agents.

## 2 Materials and methods

### 2.1 Purification and synthesis of dermaseptins

Peptides were prepared by stepwise solid phase synthesis using Fmoc polyamideactive ester chemistry on a Milligen 9050 pepsynthesizer. All Fmoc-amino acids were from Milligen/Bioresearch (Paris, France). The 4-(Hydroxymethyl) phenoacetic acid-linked polyamide/kieselguhr resin (pepsin kA), Fmoc-aminoacid pentafluorophenyl (Pfp), and 3-hydroxy-2,3-dehydro-4-oxo-benzotriazine (Dhbt) esters were given by Milligen/Bioresearch (France). Using a mixture of trifluoroacetic acid, para-cresol, thioanisol, water, and ethyl methyl sulfide (82.5 %, 5.5 %, and 2.5 % (v/v)), 5 mg of peptidyl-resin were cleaved and side chain deprotected for 2 h at room temperature. Once the resin and ether extraction were removed from the peptides, they were purified using a combination of preparative high performance liquid chromatography (HPLC), ion exchange chromatography, and Sephadex gel filtration. The homogeneity of synthesized peptides was assessed by mass spectrometry, solid phase sequence analysis, analytical HPLC, and amino acid analysis [18]. Every peptide was maintained frozen in stock solutions at a temperature of −20 °C, with a concentration of 3.5 mM in double-distilled water.

### 2.2 Estimating the Peptide's structural and physicochemical properties

The length, molecular weight (MW), and net charge (Z) of our peptides were calculated using the BACHEM peptide calculator. To calculate hydrophobicity (H) and hydrophobic moment (μH), Heliquest software was utilized [28]. An aqueous solution's general tendency of aggregation was predicted using TANGO software [29]. Although each peptide's helicity (α-helix%) was determined using AGADIR software [30].

### 2.3 Inoculum and bacterial strain authentication

The bacterial strains utilized in this investigation were donated by the microbiology laboratory of the Federal University of the Delta of Parnaíba - UFDPar, Parnaíba - PI, Brazil. The bacteria utilized in the studies were *Klebsiella pneumoniae* ATCC 700603 (producing ESBL: Extended-Spectrum Beta-Lactamase), *Staphylococcus epidermidis* 70D - MRSE (Methicillin Resistant), and *Klebsiella pneumoniae* (producing ESBL and NDM: New Delhi Metallo-beta-Lactamase). The recommended strains were grown on Mueller-Hinton agar Petri dishes (DifcoTM, Brazil) before any research was carried out. After that, they were incubated in an aerobic bacteriological incubator for 24 h at 35 ± 2 °C. After 24 h incubation, well-isolated colonies were collected using a sterile bacteriological loop and suspended in sterile saline (0.85 % NaCl) to reach a 0.5 McFarland standard (∼1–2 × 10^8^ CFU/mL), in accordance with CLSI guidelines [31]., with an absorbance pattern between 0.08 and 0.13 at 625 nm in a UV–vis spectrophotometer (Shimadzu, Japan). After the bacterial suspension was collected, it was standardized and used to create the bacterial inoculum required to complete the MIC determination procedures [31].

### 2.4 Antibacterial activity of dermaseptins

The broth microdilution technique was used to evaluate the antibacterial properties of peptides and calculate the minimum inhibitory concentration (MIC), in accordance with the criteria supplied by CLSI [31]. Using a 96-well microdilution plate (KASVI, Brazil) in contrast to the bacterial strains, the antibacterial impact was investigated. That being said, 5 μL of each peptide was added to the first line of the microtiter wells that were previously filled with 195 μL of Mueller-Hinton (M − H) broth (Life Technologies, USA). Subsequently, there were two-fold serial dilutions, resulting in final peptide concentrations ranging from 25 μg/ml to 0.19 μg/ml. At the start of the experiment, 50 μL of the bacterial inoculum was added to the test wells containing M − H broth in order to reach a final volume of 100 μL and a final concentration of 5 × 10^5^ CFU/ml. Once the serial dilution process was completed, the leftover 100 μL was disposed of. After a 24-h incubation period at 37 °C in an aerobic environment, the MIC of an antibacterial agent is defined as the absolute lowest concentration expressed in μg/ml that, under closely monitored *in vitro* conditions, effectively suppresses detectable bacterial growth. As a supplementary test to the MIC, the MBC identified the lowest dose of an antimicrobial agent that prevented the growth of bacterial colonies on the agar. In order to confirm MBC, 10 μL of the wells showing values that were either greater or equal to the MIC were seeded onto Mueller-Hinton Agar (MHA) (Life Technologies, USA) using a Drigalski spatula. Each test was conducted three times. The instructions recommended manipulating the bacterial strains under aseptic conditions in order to guarantee the safety and caliber of the procedures employed in this study. Additionally, all procedures related to the execution of the experimental protocols were carried out in a class II B2 biological safety cabinet situated in Bussettos, MG, Brazil.

### 2.5 Cell proliferation assay

The HeLa marker chromosomes were present in the HEp-2 cell line, which was acquired from HeLa contamination. It was donated by the American Type Culture Collection (ATCC, USA). Cells were routinely maintained in a humidified atmosphere with 5 % CO_2_ at 37 °C. Additives to the culture medium were 10 % (v/v) heat-inactivated fetal bovine serum (FBS), 1 % l-glutamine, and 1 % penicillin/streptomycin (Biofaster, Tunisia).

### 2.6 MTT test and cytotoxicity evaluation

The cytotoxicity test is based on assessing the ability of grown cells to survive exposure to the peptides being studied. To evaluate cytotoxicity in cultured cells (HEp-2 lines), the 3-(4, 5-dimethylthiazol-2-yl)-2, 5-diphenyl tetrazolium bromide (MTT) colorimetric test was employed. To achieve a final concentration of 1 mg/ml, distilled water was used to dilute the lyphosized peptides. The different peptides were then prepared as ½ dilutions in Eppendorf tubes using the freshly prepared medium, DMEM. There was a range of concentrations from 100 μg/ml to 1.5 μg/ml. HEp-2 cells in suspension (MEM medium with 10 % FCS) from Biofaster, Tunisia, were added to each of the 96-well plates, which held 10^5^ cells each, at a volume of 100 μl. upon that, the plates were incubated at 37 °C for 24 h with 5 % CO_2_. Once the media was withdrawn, 50 μl of DMEM medium containing 2 % FCS was added in its place. 50 μl of each peptide was placed in into every well, and using a series of 1/2 dilutions, the concentration was progressively raised from 100 μg/ml to 1.5 μg/ml. The examination was given in duplicate three times. Untreated cells will serve as the negative control. The supernatant was collected after a 72-h incubation period, and the MTT method was used to assess how vital the peptide-treated cells were. Each person added 50 μl of the MTT solution (5 mg/ml) for a brief period of time. After incubating the formazan trained crystals for 4 h at 37 °C, 100 μl of DMSO was employed to remove them, and an ELISA reader (Multiskan EX, Labsystems, France) was used to detect the optical density (OD) at 570 nm. The outcomes were expressed using the formula below: (DO544 nm peptide/DO544 nm control) × 100 is the viability percentage. The findings were shown as a percentage of viability in comparison to the untested negative control peptide. The HEp-2 CC_50_ values were subjected to half maximum cytotoxic dosages, which were calculated using GraphPad Prism® (version 9.0). The proportion of viable HEp-2 cells obtained from the findings was reported.

### 2.7 Analytical statistics

GraphPad Prism 9.0 software (GraphPad software Inc.) was used to perform the statistical analysis for the cell viability studies. The distinctions were considered statistically significant when p < 0.05.

## 3 Results and discussion

### 3.1 Conception of dermaseptin S4, B2, and their derivatives

The S4 and B2 regions of the peptides under investigation underwent deletions and/or substitutions, making three main structural changes (Table 1). By replacing the methionine (M) at position 4 with a lysine (K) and the asparagin at position 20, respectively, a derivative known as K4K20S4 was produced, with the resulting compounds being designated M4K and N20K. In peptides K4S4(1–16), the M4K change is identical, but 12 C-terminal residues are removed. It was possible to produce the K3K4B2 derivative by twice replacing a lysine (K) at position 3 (W3K) for a tryptophan residue (W) and a lysine (S) at position 4 (S4K) of B2. The one-letter code was used to ascertain the amino acid sequence of the native peptides, DRS-S4 and DRS-B2, using their length and sequence as a guide (Table 1). The initial alteration procedure aimed to increase the peptide's hydrophilicity and decrease its hydrophobicity. Our first objective in modifying this peptide was to enhance its hydrophilic characteristics by including basic amino acids such as lysine. As S4 and B2 showed some degree of biological activity with cationic residues, the choice of lysine was additionally selected to reduce an increase in cytotoxicity. In fact, it has been shown that DRS-S4's hemolytic activity was lowered whereas its net positive charge went up and its hydrophobicity decreased, all while maintaining strong biological activity [23,32,33].

**Table 1:** Dermaseptins and their analogs: Structural and Physicochemical Characteristics.

Peptides	Sequence^a^	Parameters^b^						
		Length	MW	Net Charge	H	Aggregation	μH	α-Helix %
S4 (Native)	ALWMTLLKKVLKAAAKAALNAVLVGANA	28	2.850	+4	0.544	183.33	0.248	16.55
K_4_K_20_S4	ALWKTLLKKVLKAAAKAALKAVLVGANA	28	2.861	+6	0.451	112.02	0.246	11.8
K_4_S4(1–16)	ALWKTLLKKVLKAAAK	16	1.782	+5	0.426	0	0.526	2.41
B2 (Native)	GLWSKIKEVGKEAAKAAAKAAGKAALGAVSEAV	33	3.181	+3	0.199	9.681	0.204	10.02
K_3_K_4_B2	GLKKKIKEVGKEAAKAAAKAAGKAALGAVSEAV	33	3.164	+5	0.072	9.681	0.159	9.85

a The amino acid codes are represented by a single letter in the sequences.

b Parameters: H: Hydrophobicity; MW (kDa); Aggregation: Total Aggregation Trend:; μH: Hydrophobic Moment; Helicity: α-Helix %.

**Inclusion criteria:** Peptides were selected for modification if they contained hydrophobic residues contributing to high aggregation or cytotoxicity and if their N- or C-terminal regions were known to influence antimicrobial activity.

**Modification strategy:** Lysine substitutions were introduced to increase net positive charge and hydrophilicity while reducing hydrophobicity, thereby enhancing selectivity toward bacterial membranes and decreasing toxicity toward eukaryotic cells.

**Exclusion criteria:** Peptides with fewer than 13 amino acids were excluded from certain modifications due to previously reported reduced antimicrobial efficacy. This clarification ensures that the choice of substitutions and truncations is rational, reproducible, and based on prior literature. The S4 sequence is shortened by removing 12 amino acids from the secondary structure. Previous research indicates that the long peptide lengths, low efficacy, instability, systemic toxicities, and propensity to compromise the host's natural defensive mechanisms of AMPs make them challenging to utilize as therapeutic agents. These problems have hindered the research and pharmacological implementation of AMPs [34]. Many methods for creating comparable peptides have been devised to get around these obstacles. These methods include motif hybridization, which aims to increase antimicrobial effectiveness and functionality [35]; truncation/substitution, which attempts to reduce toxicity [36]; and de novo design, which strives to decrease peptide length and remove host defense immunogenicity [37]. Previous studies have shown that whereas the N-terminal domain of DRS peptide exhibits selectivity in its interaction with the bacterial cell membrane, the C-terminal helix predominantly suggests nonspecific membrane lytic activity [38,39]. Shorter peptides between 16 and 19 mers nevertheless exhibit similar antibacterial effects, according to prior studies on DRS N-terminal peptide fragments. Nevertheless, shorter sequences containing fewer than 13 amino acid residues have far less antibacterial action [40,41]. In order to specify the structural prerequisites for biological activity, the study of peptide selection criteria was conducted. To the best of our knowledge, B2 is the derivative K3K4B2's initial use. Actually, the primary modification made to the original B2 molecule was truncation. The synthesis of the C-terminally shortened analog known as [[1], [2], [3], [4], [5], [6], [7], [8], [9], [10], [11], [12], [13], [14], [15], [16], [17], [18], [19], [20], [21], [22], [23]]-DRS-B2 is a great example of this approach. Although this reduced form maintained the net cationic charge of the parent peptide B2, it was shown to be inactive against bacteria [27]. When DRS-B2 and alginate nanoparticles (Alg NPs) are mixed, a new formulation known as “Alg NPs + DRS-B2” is created. This formulation has a significant antibacterial activity against strains of *Escherichia coli* that are sensitive to and resistant to colistin. The antibacterial activity of this new formulation is greater than that of DRS-B2 when administered alone [26]. The advantage of our synthesized peptides is that they are d-amino acid configured. On the other hand, l-amino acids, which make up peptides in their native state, provide a challenge since proteases may readily break them down. Their poor stability therefore limits their medicinal potential [42]. It is possible to overcome these restrictions by replacing the l-amino acids in the most susceptible site with d-amino acids, as previous research has demonstrated [43]. It is clear that the original peptide's net positive charge is unaffected by the addition of d-amino acids. Nevertheless, it has an impact on the elements and processes involved in chiral target identification [44,45].

### 3.2 Peptide structures and physical characteristics

**Fig. 1:**
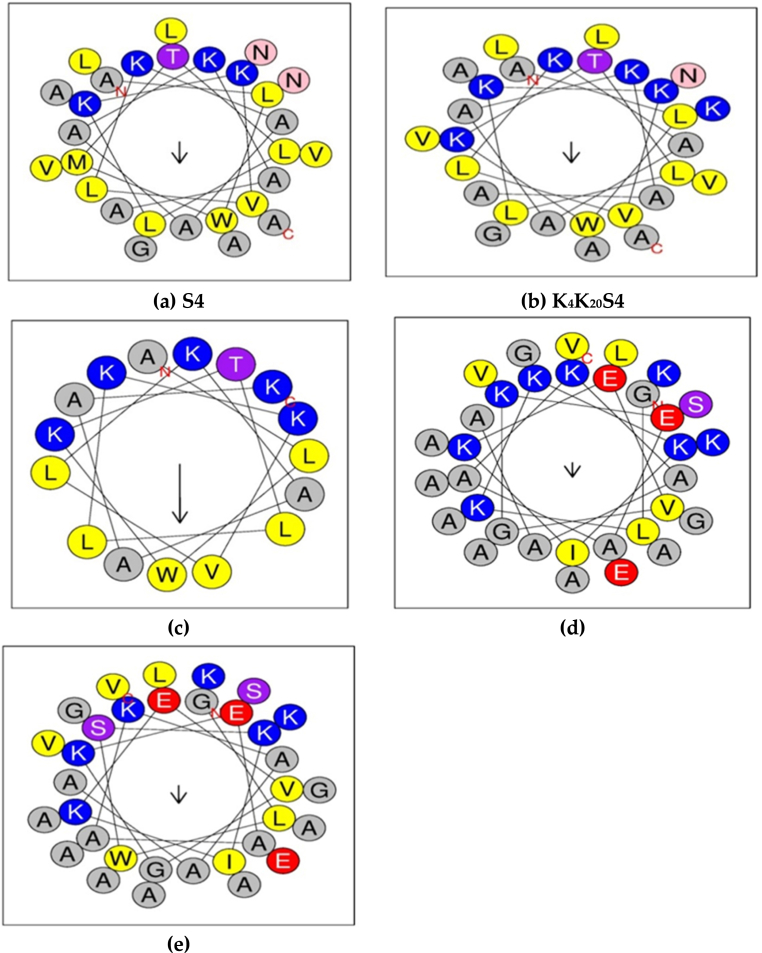
Dermaseptin helical structures and their analogs. The representation of these peptides was the two-dimensional axial projection of a symmetrical α-helix. (A) The native S4's helical structure; (B) K_4_K_20_S4's helical structure; (C) K_4_S4(1–16)'s helical structure; (D) K_3_K_4_B2's helical structure; and (E) the native B2's helical structure. The one-letter coding for amino acids is used in the figures. Figure produced by Gautier et al. (2008) with Heliquest software.

Of the S4 peptide analogs, K_4_K_20_S4 had the greatest Hydrophobicity (H) value (0.451), as determined by Heliquest, yet K_4_S4(1–16) had the lowest value (0.426) (Table 1). Overall, the H values of all DRS-S4 peptide analogs were lower than those of the original peptide (S4). Furthermore, the fact that the derivative of DRS-B2 has a higher H value (0.199) than K_3_K_4_B2 (0.072) lends greater credibility to this. The hydrophobicity of peptides is a crucial physicochemical characteristic. To find out, the peptide's sequence is often analyzed [46]. Their stability, modifications to conformation, and molecular interactions are all significantly impacted by it [47]. K_4_S4(1–16) exhibited the highest μH = 0.526 value, whilst K_3_K_4_B2 had the lowest μH = 0.159 value. The range of the μH was 0.159–0.526 (Table 1). To compute the hydrophobic moment μH, the vector average of hydrophobicity data for each amino acid alone is used [28,48]. Peptides' interfacial attachment to the membrane is significantly influenced by this aspect [49]. The TANGO algorithm [29] indicates that DRS-S4 has a greater propensity to aggregate than its derivatives because of the coupling of two hydrophobic domains at the N and C extremities (Table 1, Fig. 1). These results corroborated those of Feder et al., who demonstrated that in DRS-S4 and its derivatives, hydrophobic contacts facilitate aggregation. In fact, the M4KN20K change in the N- and C-terminal domains did reduce the aggregation propensity of K_4_S4, as per other studies [23,50]. However, when the S4 C-terminal domain is removed and positive charges are added to these areas, the aggregation decreases. K_4_(1–16)S4 had an aggregate value of zero, per Table 1. Therefore, the removal of the hydrophobic domains and/or the insertion of positive charges in these areas will probably limit the aggregation, either by the lack of such hydrophobic regions or by electrostatic repulsion among cationic residues [23,51]. These results showed that peptide aggregation is influenced by both the hydrophobicity and charge distribution of peptides. Consequently, aggregation is negatively impacted by the insertion of cationic amino acids into hydrophobic domains or by the removal of hydrophobic domains. Given that S4 exhibited a significantly higher degree of aggregation than did the natural molecule (9.681), we found that neither deletion nor substitution resulted in a decrease or loss of aggregation with respect to DRS-B2 (Table 1). Previous research has demonstrated that the tendency to aggregate in aqueous solution is another important element regulating antibacterial activity and cell selectivity [52]. The ability of peptides to form oligomers in aqueous solution and make hydrophobic connections with other monomers can account for this property. Polysaccharide capsules, outer membranes, and bacterial cell walls prevent these aggregates from interacting with the action target, the plasma membrane, because of their larger size and reduced flexibility. Consequently, peptides that have a high propensity to aggregate have a limited ability to combat bacteria; on the other hand, a monomeric peptide can more readily traverse the plasma membrane and display powerful antibacterial activity [53]. Thus, in designing antimicrobial peptides, it is often better to restrict aggregation to enhance antibacterial activity [33,53]. During our analysis, we found that all of our peptide derivatives had a decreased ability to aggregate, suggesting that they may have biological properties such as antibacterial activity. During our investigation, we estimated the helicity of the peptides using the AGADIR method [30]. According to the findings, the helicity (expressed as α-helix%) of the natural molecule S4 is around eight times higher than that of K_4_S4(1–16) and similar to that of K_4_K_20_S4. Nevertheless, Table 1 demonstrates that DRS-B2 and its analog, at 10.2 % and 9.85 %, respectively, showed the same helicity. Previous research has indicated that helicity was assessed to investigate the relationships between secondary structure and the antimicrobial peptides' selectivity against microorganisms in α-helical structures [54]. Therefore, the dermaseptins' exceptional potency may be due to both the maintenance of the helical shape and a greater net positive charge [55].

### 3.3 Toxicity of dermaseptin S4, B2, and derivatives on HEp-2 cells *in vitro*

All of our peptides were examined for cytotoxicity on HEp-2 cells using the MTT viability assay. The outcomes are shown as survival rates after the compounds were administered for 72 h (Table 2).

**Table 2:** Dermaseptins and their derivatives' Antimicrobial Properties and dose-dependent Effects.

Peptides	CC_50_ Hep-2 cells (μg/ml)	*Staphylococcus epidermidis 70D* MIC (μg/ml)	*Klebsiella pneumoniae ATCC 700603* MIC (μg/ml)	*Klebsiella pneumoniae (ESBL and NDM)* MIC (μg/ml)
S4	16.51	25	25	25
K_4_S4(1–16)	68.9	12.5	12.5	12.5
K_4_K_20_S4	75.71	6.25	6.25	6.25
B2	30.4	25	25	25
K_3_K_4_B2	61.25	12.5	12.5	12.5
Meropenem	ND	32	32	32

CC_50_: peptide concentration that causes 50 % cytotoxicity in HEp-2 cells for dermaseptin S4, B2 and derivatives (μg/ml); ND, not determined; MIC: Minimum Inhibitory Concentration (μg/ml).

Our results show that the cationic analogs under investigation were found to be toxic to HEp-2 cells to differing degrees. Peptides were added to the cells at concentrations varying between 1.5 and 100 μg/ml. Table 2 revealed the peptide 50 % cytotoxic concentration (CC_50_) values, and the cytotoxicity of DRSs depended on concentration (results not shown). The highest cytotoxicity values for S4 and B2 derivative were discovered at doses higher than 61.25 μg/ml (CC_50_). Interestingly, K_4_S4(1–16) peptide's C-terminal extremity may be shortened to provide low-toxicity peptides (CC_50_ of around 75.71 μg/ml and 61.25 μg/ml, respectively), and it can be positively charged by substituting K_4_K_20_S4 or K_3_K_4_B2. Belaid et al. reported the highest non-cytotoxic doses of DRS-S1, DRS-S2, 64 μg/ml of dermaseptin S5, and 16 μg/ml of DRS-S3 and DRS-S4 to HEp-2 cells [56]. Moreover, Lactoferricin (17–30)a and Cecropin A (1–7)-Melittin, two other AMPs, demonstrated restricted cytotoxicity at lower doses (1X and 2X MIC); at 4X MIC, nonetheless a small improvement in cytotoxicity was observed, taking into account that the MIC values are 64 μg/ml and 128 μg/ml. This information is supported by Gourkhede et al. study [57]. Accordingly. A study by Sruthy et al. [58] found that the highest tested dosage of histone H2A-derived antimicrobial peptide (200 μM) resulted in 89 % reduced proliferation of HEp2 cell lines. The cytotoxic effects of DRS-B2 and the novel formulation (Alg NPs + DRS-B2) on human erythrocytes and the eukaryotic cell line types IPEC-1 (animal) and HT29 (human) were assessed by Hazime et al. [26]. Both compositions' safety was verified. Zairi et al. [59] reported that dermaseptin K_4_S4 exhibited a more powerful toxicity profile when administered to human endometrial epithelial cells, which exhibited a lower susceptibility to the adverse effects of dermaseptins in comparison to other cell types. Although their cellular selectivity or method of action are yet unknown, dermaseptin S4 and its derivatives exhibit significant cytotoxicity against the SW620 cell line [60]. Additionally, it was demonstrated by Lorin et al. [61] that dermaseptin K4_S_4(1–16)a was non-toxic in mice, had diminished cell toxicity at high doses, and had a comparable effect on primary PBMCs and HeLa P4-CCR5 cells. All of this study has shown that generated and altered peptides are less harmful to HEp-2 cells than the original molecules S4 and B2.

### 3.4 Antibacterial activity of dermaseptin derivatives on *K. pneumoniae* and *S. epidermidis*

Bacteria susceptibility to DRS-S4 and DRS-B2 derivatives has been evaluated by measuring the peptide MIC against *Staphylococcus epidermidis* 70D – MRSE, a clinical (resistant) strain, *Klebsiella pneumoniae* ATCC 700603, a sensitive strain that produces ESBL, and *Klebsiella pneumoniae*, a clinical (resistant) strain that produces ESBL and NDM. The strain that generates NDM and ESBL is far more resistant to antibiotics than the variant that produces ESBL. Table 2 contains the data that were gathered. Given the structure of the peptide, the findings show that all peptides examined, with highly charged peptides being the most powerful, inhibited the growth of both Gram-positive and Gram-negative bacteria. 6.25–25 μg/ml was the range of MICs. Thus, demonstrating the potential of these peptides as antimicrobials. The most active peptide is K_4_K_20_S4, as Table 2 illustrates. This particular peptide exhibited potent antibacterial action, as seen by its MICs of around 6.25 μg/ml. Likewise, the essentially homogenous potency and 12.5 μg/ml MIC of the mono-substituted shortening peptide K_4_S4(1–16) are noteworthy. If the C-terminal extremity of the peptide is removed, it becomes less toxic and still performs. The C-terminal domain also contributes to binding affinity, as suggested by Kustanovich, while the N-terminal domain's attachment to the cell membrane is primarily dependent on the net charge state [33]. Despite being less efficient against all tested strains, the natural DRS-S4 exhibited the same MIC value as the original B2, measuring at 25 μg/ml. The analog K_3_K_4_B2 is likewise more active than the original B2, comparable to K_4_S4(1–16). MIC for the peptide is 12.5 μg/ml. An increase in positive charge is required to improve the antibacterial properties of both DRS-S and DRS-B analogs. K_4_K_20_S4 and K_3_K_4_B2 are consequently the most potent analogs against clinical strain *S. epidermidis* 70D and both resistant and sensitive strains of *K. pneumoniae* because they have the lowest MIC values. Our results show that native substances under the same circumstance, such as B2 and S4, remain less active than their cationic analog.

The clinical (resistant) strain *Staphylococcus epidermidis 70D - MRSE*, the sensitive strain *Klebsiella pneumoniae ATCC 700603* and the clinical strain *Klebsiella pneumoniae* that produces both ESBL and NDM, were used to evaluate the MBC following the determination of the MIC. An antibiotic agent called meropenem was used. One negative control was a solvent that had been saturated with saline. Conversely, the reference medication meropenem had a MIC of 32 μg/ml and an MBC value of 64 μg/ml and above. In addition to having minimal toxicity, meropenem is a beta-lactam antibiotic belonging to the carbapenem class. Since it efficiently combats a broad variety of bacteria, this antimicrobial agent is a beneficial and often recommended therapy for serious and nosocomial infections [62]. The microorganisms that Meropenem targets are: anaerobic, Gram-positive, and Gram-negative. By interfering with the production of bacterial cell walls, meropenem suppresses bacterial growth and induces cell death in the same way as other carbapenems [63]. Treatment failure and the potential for the emergence of microbial resistance are two consequences of meropenem concentrations below therapeutic limits [64].

MBC showed findings for both dermaseptin derivatives that ranged from 12.5 μg/ml to 50 μg/ml, either exceeding or equaling the MIC values. Against *S. epidermidis* 70D-MRSE (Gram +), the highest MBC (50 μg/ml) displayed the native molecules S4 value. K_4_K_20_S4 has MBC (6.25) that is the lowest. For *Klebsiella spp.* (Gram -) in K_4_S4(1–16) and K_3_K_4_B2, MBC is equal to 12.5 μg/ml, and nearly twice as much, at 25 μg/ml, in *S. epidermidis*. Table 2 shows the identical MIC values for the two Gram+ and Gram-strains of bacteria, while Table 3 shows that the MBC values for susceptible and resistant strains of *K. pneumoniae* are twice as high as those for *S. epidermidis*. One important characteristic of infecting isolates is methicillin resistance in *S. epidermidis* (MRSE), as it is often associated with other mechanisms of antibiotic resistance [9].

**Table 3:** Minimal Bactericidal Concentration (MBC) of S4, B2 and their analogs against multidrug resistant bacteria.

Peptides	*Staphylococcus epidermidis 70D* MBC (μg/ml)	*Klebsiella pneumoniae ATCC*700603 MBC (μg/ml)	*Klebsiella pneumoniae (ESBL and NDM)* MBC (μg/ml)
S4	50	25	25
K_4_S4(1–16)	25	12.5	12.5
K_4_K_20_S4	12.5	6.25	6.25
B2	50	25	25
K_3_K_4_B2	25	12.5	12.5
meropenem	64	64	64

MBC: Minimal Bactericidal Concentration (μg/ml).

However, Chen et al. [65] have already demonstrated the antibacterial efficacy of Dermaseptin-AC against *K. pneumoniae* ATCC 43816. The findings indicated that the MBC was 20.488 μg/ml and the MIC was 5.122 μg/ml. These results are in general consistent with what we succeeded to achieve. Studies reveal that *K. pneumoniae* secretes capsular polysaccharides that act as a barrier for AMPs, changing their structure and preventing them from interacting with microbial membranes [66]. SYTOX Green assay findings showed that dermaseptin-AC enhanced *K. pneumoniae*'s cell membrane permeability at 4 MIC. Chen et al. [65] found Dermaseptin-AC4 (SLWGKLKEMAAAAGKAALNAVNGLVNQ-NH2) as another AMP from *A. callidryas* skin secretions. Two synthetic peptides, Dermaseptin-AC4a and Dermaseptin-AC4b, were named after Dermaseptin-AC4 [67]. *In vitro* antibacterial activity against *K. pneumoniae* was much lower for Dermaseptin-AC4 and its analogs than for Dermaseptin-AC. This disparity might be explained by the fact that Dermaseptin-AC is more hydrophobic, positively charged, and more susceptible to forming an α-helical shape when in contact with the cell membrane. Compared to Dermaseptin-AC, the hemolytic activity of Dermaseptin-AC4 and its derivatives was lower. In spite of this, the MIC and MBC of Dermaseptin-AC4a were higher than those of Dermaseptin-AC. These results support our study, which demonstrates that charge is a critical component in enhancing the bioactivity of antimicrobial peptides [68]. Li et al. [69] isolated and identified dermaseptin-PT9 (DPT9), a new dermaseptin peptide, from *Phyllomedusa tarsius*. The MIC and MBC values of this peptide against *K. pneumoniae* ATCC 43816 were 20.8 μg/ml and 41.7 μg/ml, respectively, indicating its effectiveness. Furthermore, K^8^, ^23^-DPT9, an improved cationicity counterpart, exhibited a significantly higher antibacterial action against the same strain, with a MIC of 5.2 μg/ml and an MBC of around 10.4 μg/ml, as well as disrupting microbial cell membranes [68]. This was achieved by the substitution of lysine residues for Asp8 and Glu23. Both peptides demonstrated similar effects on membrane permeabilization when exposed to *K. pneumoniae*. The results indicated that while there was significant membrane permeabilization at each peptide's MIC, full or almost entire membrane rupture was observed at dosages equal to four times the MICs [68]. Dermaseptin-PP from *Phyllomedusa palliata*, a rarely studied frog species, was found by Dong et al. [70] to have broad-spectrum antibacterial activity against *K. pneumoniae* (ATCC 43816) at values of MIC = 1 μM and MBC = 2 μM, as well as a relatively low hemolytic impact (estimated HC50 = 38.77 μM).

The antibacterial activity of DRS-B2 and DRS-S4, as well as their derivatives, against *S. epidermidis* and *K. pneumoniae*, have never before been assessed, as far as we are aware of. According to earlier studies, DRS-B1 and S1 are effective against both gram-positive and gram-negative bacteria *in vitro* and have varying specificities [71]. Derivatives of DRS-S4, DRS-CA1, DRS-DU1, and DRS-PH also show *in vitro* activity against *Pseudomonas aeruginosa*, *E. coli*, and *Staphylococcus aureus* (including the methicillin-resistant strain), according to scientific literature [40,[72], [73], [74]]. This is also the case when they form a biofilm. In accordance with Zairi et al. [72], DRS-S4 analogs are less cytotoxic than conventional antibiotics.

Other AMPs have been demonstrated to be effective against *S. epidermidis*, despite the lack of research assessing our dermaseptins' antibacterial efficacy against this strain of bacteria in the literature. It has been shown that temperin A works against both methicillin-susceptible and methicillin-resistant strains of *S. epidermidis* bacteria by inhibiting the formation of biofilms [75]. Human erythrocytes were unaffected, and its MIC was 8 mg/l. Temporin A may function by causing the bacterial murine hydrolases to break down the peptidoglycan layer and cause cell lysis, or it can behave by creating pores or channels [75]. While temperin-1DRa is cytotoxic to mammalian cells, it is also very effective against *S. epidermidis*. Changes in cationicity increased antimicrobial potential, while changes in hydrophobicity and helicity decreased hemolytic effect and contributed to nontoxic peptide, according to a comprehensive analysis of the effects of altering amino acid residues on cytotoxicity and antimicrobial activity [75,76]. In order to attack *S. epidermidis*, certain naturally occurring peptides have been modified and found to function better when combined. Royal Jellein I, II, and III—which were modified at the C-terminal to become RJ I–C, II-C, and III-C, respectively—are secreted by the honeybee *Apis mellifera*. These peptides folded and accumulated inside the membrane, acting as antistaphylococci [75]. Other polycationic peptides that have shown antibacterial activity against *S. epidermidis* include buforin II and ranalexin, which were extracted from the skin of an American bullfrog and the stomach tissue of an Asian toad, respectively [77]. It has been noted that de novo-designed AMPs, natural peptides, and their derivatives have antibacterial and antibiofilm action against *S. epidermidis*. These peptides function in a variety of ways. First, they might interfere with the targeted cell's nucleic acids, or they can disrupt the lipid bilayer to target bacterial cell membranes [75].

Evaluating the peptide-membrane interaction and resistance mechanisms of *K. pneumoniae* and *S. epidermidis* is essential to developing new antimicrobial drugs or other approaches to address this serious public health issue. Some of the main categories into which the mechanisms of drug resistance may be categorized include drug inactivation or alteration, modification of drug binding sites or targets, changes in cell permeability leading to decreased intracellular drug accumulation, and the formation of biofilms [78]. The primary reported interaction between AMPs and the bacterial cytoplasmic membrane is that it modifies both the membrane's integrity and electrical potential [79]. Amphipathic molecules (AMPs) and their effects on the membrane are determined by the properties of the peptides, such as size, structure, net charge, amphipathicity, and composition [80]. AMPs cannot alter the cytoplasmic membrane or operate on Gram-negative bacteria in any other manner until they permeate the two membrane layers that make up these bacteria during their contact stage [81]. When AMPs interact with the membrane, they may also have an impact on the *trans*-membrane voltage, sometimes referred to as the membrane potential. Cell division, membrane transport, and ATP synthesis are typically controlled by the membrane potential [81]. On the other hand, Jiang et al. [82] suggest that AMPs interact with negatively charged bacterial cell membranes by a process known as the "carpet mechanism," which is comparable to how a detergent functions [83]. This technique enables antibacterial activity to occur without requiring the insertion of a tans membrane [82]. The way that AMPs disrupt cells proceeds in three primary stages. During the attraction phase, the cationic peptides interact with the bacterial surface. The cationic peptide adheres to the bacterial outer membrane surfaces by means of lipopolysaccharide as a receptor. Due to their positive charge, AMPs may divide the outer membrane and reach their internal target, which are negatively charged phospholipids, by displacing cationic ions such as Ca2+ and Mg2+ upon attachment [84,85]. The second phase is attachment, which calls for the penetration, attachment, and interaction of polysaccharides with biological targets, such as the protective capsular coats of gram-positive or gram-negative bacteria, or their teichoic and lipoteichoic acids [84]. Following their passage through the cell membrane, AMPs interact with phosphatidylglycerol and cardiolipin in the cytoplasmic membrane to produce permeability or disintegration by weakening their bonds [86].

### 3.5 Structure-function relationship

**Fig. 2:**
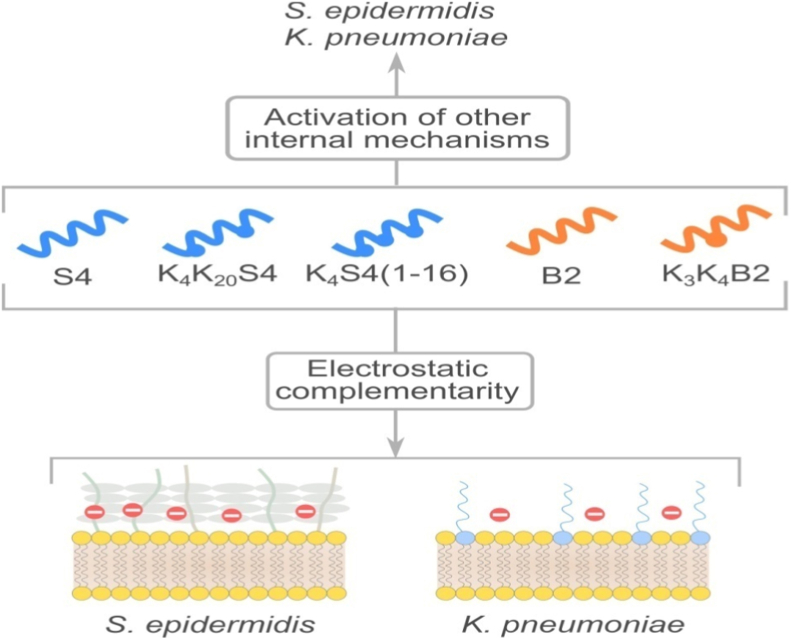
The structure-function relationship of the dermaseptin S4 and B2 and their analogs interacting with *S. epidermidis* and *K. pneumoniae* membranes.

When interacting with host cells, peptides can display different reactions that are dictated by the electrostatic properties of both the peptide and the target cell (Fig. 2). This is apparent from the charge complementarity between the cationic peptides we investigated at and the membrane of the negatively charged bacteria we studied. The greatest electropositivity is shown in the N-terminal region of S4 and its derivatives, indicating that this region is crucial in determining the biological activity. The bactericidal and inhibitory activities of DRS-S4, DRS-B2, and their derivatives against all studied bacterial strains (both Gram-positive and Gram-negative) provide proof of this. Zeta potential measurements revealed values of −17 mV for *S. epidermidis* and −10 mV to −26 mV for *K. pneumoniae* [87,88]. These results are consistent with the charge complementarity between the target bacteria and the AMPs. The charge effect appears to improve dermaseptins' inhibitory capacities against Gram-negative bacteria, however not their bactericidal properties to the same degree. This is noteworthy. The zeta potential's approximation of the electrostatic interactions between the peptides and the bacterial membrane is supported by the similar MIC values for *K. pneumoniae* and *S. epidermidis*. The MBC values, however, suggest that the bactericidal effect might not be directly connected with the electrostatic interactions: In comparison to both sensitive and resistant strains of *K. pneumoniae*, MBC for *S. epidermidis* is twice as high. This suggests that there may be other intracellular processes that the peptides stimulate, prompting the need for more research. This observation challenges the initial hypothesis that the dense peptidoglycan layers may act as a barrier for the peptide to reach the phospholipid membrane, a notion that has been previously shown to be inaccurate [89]. It is consistent with the significant difference in MBC between Gram-positive and Gram-negative bacteria.

## 4 Conclusion

According to the study's findings, *Klebsiella pneumoniae* and *Staphylococcus epidermidis* are significantly and specifically inhibited by our dermaseptines belonging to the S4 and B2 families. This study also demonstrated the concentration-dependent cytotoxicity of these modified peptides. Based on our investigation, the most promising candidate against antibacterial activity is the bi-substituted peptide K_4_K_20_S4. It is the most frequent net positive charge (+6), has the largest CC_50_ (75.71 μg/ml), and has lower values of MIC (6.25 μg/ml) and MBC (12.5 μg/ml against *S. epidermidis* and 6.25 μg/ml against both sensitive and resistant strains of *K. pneumoniae*). When combined, these minor elements have the potential to produce safe antibacterial compounds.

Taken together, this study provides the first evidence of antibacterial and cytotoxic activities of newly designed dermaseptin B2-derived peptides, including the K3K4B2 variant, against *Staphylococcus epidermidis* and *Klebsiella pneumoniae*. The originality of this work lies in the rational design of novel B2-based derivatives, which had not been previously reported or tested against these clinically relevant multidrug-resistant strains. Future work will evaluate the efficacy and safety of the most promising dermaseptin derivatives *in vivo*, optimize peptide stability and pharmacokinetics, and explore combinations with nanoparticles or conventional antibiotics to enhance antimicrobial activity and reduce cytotoxicity, aiming to translate *in vitro* findings into preclinical applications.

## Institutional review board statement

Not applicable.

## Funding statement

This research received no external funding.

## CRediT authorship contribution statement

**Houda Haddad:** Conceptualization, Methodology, Writing – original draft. **Reyadh R. AL-Rashidi:** Conceptualization, Methodology. **Ahmed Loghmari:** Validation, Writing – review & editing. **Wissal Sahtout:** Validation, Writing – review & editing. **Raja Boukadida:** Methodology, Validation. **Rihem Dahmene:** Methodology, Validation. **Emeny Ettouil:** Investigation, Methodology. **Houcemeddine Othman:** Methodology. **Ines Ouahchi:** Formal analysis. **Amira Zaϊri:** Supervision, Writing – review & editing.

## Declaration of competing interest

The authors declare that they have no known competing financial interests or personal relationships that could have appeared to influence the work reported in this paper.

## Data Availability

Materials, data, and associated protocols are available to readers without undue qualifications regarding material transfer agreements.

## References

[1] Demiankova M.V., Giovannercole F., Khomutov M.A., Salikhov A.I., Onillon L., Valuev-Elliston V.T., Vasilieva B.F., Khurs E.N., Gabrielyan N.I., Kochetkov S.N. (2023). Antibacterial activity of peptide derivatives of phosphinothricin against multidrug-resistant *Klebsiella pneumoniae*. Molecules.

[2] Garrity G.M., Brenner D.J., Krieg N.R., Staley J.T. (2005).

[3] Podschun R., Pietsch S., Höller C., Ullmann U. (2001). Incidence of *Klebsiella* species in surface waters and their expression of virulence factors. Appl. Environ. Microbiol..

[4] Krawczyk B., Wysocka M., Michalik M., Gołębiewska J. (2022). Urinary tract infections caused by *K. pneumoniae* in kidney transplant recipients - epidemiology, virulence and antibiotic resistance. Front. Cell. Infect. Microbiol..

[5] Asri N.A.M., Ahmad S., Mohamud R., Hanafi N.M., Zaidi N.F.M., Irekeola A.A., Shueb R.H., Yee L.C., Noor N.M., Mustafa F.H. (2021). Global prevalence of nosocomial multidrug-resistant *Klebsiella pneumoniae*: a systematic review and meta-analysis. Antibiotics.

[6] Galani I., Karaiskos I., Giamarellou H. (2021). Multidrug-resistant *Klebsiella pneumoniae*: mechanisms of resistance including updated data for novel β-lactam-β-lactamase inhibitor combinations. Expert Rev. Anti Infect. Ther..

[7] Santajit S., Indrawattana N. (2016). Mechanisms of antimicrobial resistance in ESKAPE pathogens. BioMed Res. Int..

[8] Ashurst J.V., Dawson A. (2022).

[9] Sabaté Brescó M., Harris L.G., Thompson K., Stanic B., Morgenstern M., O'Mahony L., Richards R.G., Moriarty T.F. (2017). Pathogenic mechanisms and host interactions in *Staphylococcus epidermidis* device-related infection. Front. Microbiol..

[10] Morgenstern M., Erichsen C., Hackl S., Mily J., Militz M., Friederichs J. (2016). Antibiotic resistance of commensal *staphylococcus aureus* and coagulase-negative staphylococci in an international cohort of surgeons: a prospective point-prevalence study. PLoS One.

[11] Widerstrom M. (2016). Significance of *Staphylococcus epidermidis* in health care associated infections, from contaminant to clinically relevant pathogen: this is a wake-up call. J. Clin. Microbiol..

[12] Pinheiro L., Brito C.I., Pereira V.C., Oliveira A., Bartolomeu A.R., Camargo C.H. (2016). Susceptibility profile of *Staphylococcus epidermidis* and *Staphylococcus haemolyticus* isolated from blood cultures to vancomycin and novel antimicrobial drugs over a period of 12 years. Microb. Drug Resist..

[13] Cherifi S., Byl B., Deplano A., Nonhoff C., Denis O., Hallin M. (2013). Comparative epidemiology of *Staphylococcus epidermidis* isolates from patients with catheter-related bacteremia and from healthy volunteers. J. Clin. Microbiol..

[14] Chu V.H. (2009). Coagulase-negative staphylococcal prosthetic valve endocarditis--a contemporary update based on the International Collaboration on Endocarditis: prospective cohort study. Heart.

[15] Bahar A.A., Ren D. (2013). Antimicrobial peptides. Pharmaceuticals.

[16] Mwangi J., Hao X., Lai R., Zhang Z.Y. (2019). Antimicrobial peptides: new hope in the war against multidrug resistance. Zool. Res..

[17] Brown K.L., Hancock R.E. (2006). Cationic host defense (antimicrobial peptides). Curr. Opin. Immunol..

[18] Mor A., Nguyen V.H., Delfour A., Migliore-Samour D., Nicolas P. (1991). Isolation, amino acid sequence, and synthesis of dermaseptin, a novel antimicrobial peptide of amphibian skin. Biochemistry.

[19] Amiche M., Ladram A., Nicolas P. (2008). A consistent nomenclature of antimicrobial peptides isolated from frogs of the subfamily *Phyllomedusinae*. Peptides.

[20] Zairi A., Tangy F., Saadi S., Hani K. (2008). *In vitro* activity of dermaseptin S4 derivatives against genital infections pathogens. Regul. Toxicol. Pharmacol..

[21] Nicolas P., El Amri C. (2009). The dermaseptin superfamily: a gene-based combinatorial library of antimicrobial peptides. Biochim. Biophys. Acta.

[22] Shai Y. (2002). Mode of action of membrane active antimicrobial peptides. Biopolymers.

[23] Feder R., Dagan A., Mor A. (2000). Structure-activity relationship study of antimicrobial dermaseptin S4 showing the consequences of peptide oligomerization on selective cytotoxicity. J. Biol. Chem..

[24] Amiche M., Ducancel F., Mor A., Boulain J., Menez A., Nicolas P. (1994). Precursors of vertebrate peptide antibiotics dermaseptin b and adenoregulin have extensive sequence identities with precursors of opioid peptides dermorphin, dermenkephalin, and deltorphins. J. Biol. Chem..

[25] Daly J.W., Caceres J., Moni R.W., Gusovsky F., Moos M., Seamon K.B., Milton K., Myers C.W. (1992). Frog secretions and hunting magic in the upper Amazon: identification of a peptide that interacts with an adenosine receptor. Proc. Natl. Acad. Sci..

[26] Hazime N., Belguesmia Y., Barras A., Amiche M., Boukherroub R., Drider D. (2022). Enhanced antibacterial activity of dermaseptin through its immobilization on alginate Nanoparticles—Effects of menthol and lactic acid on its potentialization. Antibiotics.

[27] Galanth C., Abbassi F., Lequin O., Ayala-Sanmartin J., Ladram A., Nicolas P., Amiche M. (2009). Mechanism of antibacterial action of dermaseptin B2: interplay between helix-hinge-helix structure and membrane curvature strain. Biochemistry.

[28] Gautier R., Douguet D., Antonny B., Drin G. (2008). HELIQUEST: a web server to screen sequences with specific α-helical properties. Bioinformatics.

[29] Fernández-Escamilla A.M., Rousseau F., Schymkowitz J., Serrano L. (2004). Prediction of sequence-dependent and mutational effects on the aggregation of peptides and proteins. Nat. Biotechnol..

[30] Muñoz V., Serrano L. (1994). Elucidating the folding problem of helical peptides using empirical parameters. Nat. Struct. Mol. Biol..

[31] CLSI, Clinical and Laboratory Standards Institute (2018).

[32] Efron L., Dagan A., Gaidukov L., Ginsburg H., Mor A. (2002). Direct interaction of dermaseptin S4 aminoheptanoyl derivate with intraerythrocytic malaria parasite leading to increased specific antiparasitic activity in culture. J. Biol. Chem..

[33] Kustanovich I., Shalev D.E., Mikhlin M., Gaidukov L., Mor A. (2002). Structural requirements for potent versus selective cytotoxicity for antimicrobial dermaseptin S4 derivatives. J. Biol. Chem..

[34] Ong Z.Y., Wiradharma N., Yang Y.Y. (2014). Strategies employed in the design and optimization of synthetic antimicrobial peptide amphiphiles with enhanced therapeutic potentials. Adv. Drug. Deliv..

[35] Ma Z., Wei D., Yan P., Zhu X., Shan A., Bi Z. (2015). Characterization of cell selectivity, physiological stability and endotoxin neutralization capabilities of alpha-helix-based peptide amphiphiles. Biomaterials.

[36] Lyu Y., Yang Y., Lyu X., Na D., Shan A. (2016). Antimicrobial activity, improved cell selectivity and mode of action of short PMAP-36-derived peptides against bacteria and Candida. Sci. Rep..

[37] Dong N., Zhu X., Chou S., Shan A., Li W., Jiang J. (2014). Antimicrobial potency and selectivity of simplified symmetric-end peptides. Biomaterials.

[38] Van Zoggel H., Carpentier G., Dos Santos C., Hamma-Kourbali Y., Courty J., Amiche M., Delbé J. (2012). Antitumor and angiostatic activities of the antimicrobial peptide dermaseptin B2. PLoS One.

[39] Irazazabal L.N., Porto W.F., Ribeiro S.M., Casale S., Humblot V., Ladram A., Franco O.L. (1858). Selective amino acid substitution reduces cytotoxicity of the antimicrobial peptide mastoparan. Biochim. Biophys. Acta.

[40] Navon-Venezia S., Feder R., Gaidukov L., Carmeli Y., Mor A. (2002). Antibacterial properties of dermaseptin S4 derivatives with *in vivo* activity. Antimicrob. Agents Chemother..

[41] Krugliak M., Feder R., Zolotarev V.Y., Gaidukov L., Dagan A., Ginsburg H., Mor A. (2000). Antimalarial activities of dermaseptin S4 derivatives. Antimicrob. Agents Chemother..

[42] Walter R., Neidle A., Marks N. (1975). Significant differences in the degradation of pro-leu-gly-nH2 by human serum and that of other species (38484). Proc. Soc. Exp. Biol. Med..

[43] Hong S.Y., Oh J.E., Lee K.H. (1999). Effect of D-amino acid substitution on the stability, the secondary structure, and the activity of membrane-active peptide. Biochem. Pharmacol..

[44] Braunstein A., Papo N., Shai Y. (2004). *In vitro* activity and potency of an intravenously injected antimicrobial peptide and its DL amino acid analog in mice infected with bacteria. Antimicrob. Agents Chemother..

[45] Zhao Y., Zhang M., Qiu S., Wang J., Peng J., Zhao P., Zhu R., Wang H., Li Y., Wang K. (2016). Antimicrobial activity and stability of the D-amino acid substituted derivatives of antimicrobial peptide polybia-MPI. AMB Express.

[46] Vaezi Z., Bortolotti A., Luca V., Perilli G., Mangoni M.L., Khosravi-Far R., Bobone S., Stella L. (2020). Aggregation determines the selectivity of membrane-active anticancer and antimicrobial peptides: the case of killer FLIP. Biochim. Biophys Acta. Bio..

[47] Al Musaimi O., Valenzo O.M.M., Williams D.R. (2023). Prediction of peptides retention behavior in reversed-phase liquid chromatography based on their hydrophobicity. J. Separ. Sci..

[48] Eisenberg D., Weiss R.M., Terwilliger T.C. (1984). The hydrophobic moment detects periodicity in protein hydrophobicity. Proc. Nat. Acad. Sci..

[49] Eisenberg D., Weiss R.M., Terwilliger T.C. (1982). The helical hydrophobic moment: a measure of the amphiphilicity of a helix. Nature.

[50] Dennison S.R., Phoenix D.A. (2011). Influence of C-terminal amidation on the efficacy of modelin-5. Biochemistry.

[51] Bartels E.J.H., Dekker D., Amiche M. (2019). Dermaseptins, multifunctional antimicrobial peptides: a review of their pharmacology, effectivity, mechanism of action, and possible future directions. Front. Pharmacol..

[52] Zou R., Zhu X., Tu Y., Wu J., Landry M.P. (2018). Activity of antimicrobial peptide aggregates decreases with increased cell membrane embedding free energy cost. Biochemistry.

[53] Torres M., Sothiselvam Sh, Lu T.K., de la Fuente-Nunez C. (2019). Peptide design principles for antimicrobial applications. J. Mol. Biol..

[54] Huang Y., He L., Li G., Zhai N., Jiang H., Chen Y. (2014). Role of helicity of α-helical antimicrobial peptides to improve specificity. Protein Cell.

[55] Zelezetsky I., Tossi A. (2006). Alpha-helical antimicrobial peptides—using a sequence template to guide structure–activity relationship studies. Biochim. Biophys. Acta.

[56] Belaid A., Aouni M., Khelifa R., Trabelsi A., Jemmali M., Hani K. (2002). *In vitro* antiviral activity of dermaseptins against herpes simplex virus type 1. J. Med. Virol..

[57] Gourkhede D.P., Bhoomika S., Pathak R., Yadav J.P., Nishanth D., Vergis J., Malik S.V.S., Barbuddhe S.B., Rawool D.B. (2020). Antimicrobial efficacy of Cecropin A (1-7,- Melittin and Lactoferricin (17-30, against multi-drug resistant *Salmonella Enteritidis*. Microb. Pathog..

[58] Sruthy K.S., Nair A., Antony S.P., Puthumana J., Singh I.S.B., Philip R. (2019). A histone H2A derived antimicrobial peptide, Fi-Histin from the Indian White shrimp, *Fenneropenaeus indicus*: molecular and functional characterization. Fish Shellfish Immunol..

[59] Zairi A., Serres C., Tangy F., Jouannet P., Hani K. (2008). *In vitro* spermicidal activity of peptides from amphibian skin: dermaseptin S4 and derivatives. Bioorg. Med. Chem..

[60] Belaid A., Braiek A., Alibi S., Hassen W., Beltifa A., Nefzi A., Mansour H.B. (2021). Evaluating the effect of dermaseptin S4 and its derivatives on multidrug-resistant bacterial strains and on the colon cancer cell line SW620, Env. Sci. Pollut. Res. Int..

[61] Lorin C., Saidi H., Belaid A., Zairi A., Baleux F., Hocini H., Bélec L., Hani K., Tangy F. (2005). The antimicrobial peptide dermaseptin S4 inhibits HIV-1 infectivity *in vitro*. Virology.

[62] Streit F., Perl T., Schulze M.H., Binder L. (2016). Personalised beta-lactam therapy: basic principles and practical approach. LaboratoriumsMedizin.

[63] (2007). IV. Merremrm, (Meropenem for Injection,: US Prescribing Information.

[64] Steffens N.A., Zimmermann E.S., Nichelle S.M., Brucker N. (2021). Meropenem use and therapeutic drug monitoring in clinical practice: a literature review. J. Clin. Pharm. Therapeut..

[65] Chen J., Hao D., Mei K., Li X., Li T., Ma C., Xi X., Li L., Wang L., Zhou M., Chen T., Liu J., Wu Q. (2021). *In Vitro* and *In Vivo* studies on the antibacterial activity and safety of a new antimicrobial peptide Dermaseptin-AC. Microbiol. Spectr..

[66] Guilhelmelli F., Vilela N., Albuquerque P., Derengowski L.S., Silva-Pereira I., Kyaw C.M. (2013). Antibiotic development challenges: the various mechanisms of action of antimicrobial peptides and of bacterial resistance. Front. Microbiol..

[67] Gong Z., Pei X., Ren S., Chen X., Wang L., Ma C., Xi X., Chen T., Shaw C., Zhou M. (2020). Identification and rational design of a novel antibacterial peptide Dermaseptin-AC from the skin secretion of the red-eyed tree frog *Agalychnis callidryas*. Antibiotics.

[68] Giangaspero A., Sandri L., Tossi A. (2001). Amphipathic alpha helical antimicrobial peptides. Eur. J. Biochem..

[69] Li M., Xi X., Ma C., Chen X., Zhou M., Burrows J.F., Chen T., Wang L. (2019). A novel dermaseptin isolated from the skin secretion of *Phyllomedusa tarsius* and its cationicity-enhanced analogue exhibiting effective antimicrobial and anti-proliferative activities. Biomolecules.

[70] Dong Z., Hu H., Yu X., Tan L., Ma C., Xi X., Li L., Wang L., Zhou M., Chen T., Du S., Lu Y. (2020). Novel frog skin-derived peptide Dermaseptin-PP for lung cancer treatment: *in vitro/vivo* evaluation and anti-tumor mechanisms Study. Front. Chem..

[71] Strahilevitz J., Mor A., Nicolas P., Shai Y. (1994). Spectrum of antimicrobial activity and assembly of dermaseptin-b and its precursor form in phospholipid membranes. Biochemistry.

[72] Zairi A., Ferrieres L., Latour-Lambert P., Beloin C., Tangy F., Ghigo J.M. (2014). *In vitro* activities of dermaseptins K4S4 and K4K20S4 against *Escherichia coli, Staphylococcus aureus*, and *Pseudomonas aeruginosa* planktonic growth and biofilm formation. Antimicrob. Agents Chemother..

[73] Liu J., Wu Q., Li L., Xi X., Wu D., Zhou M. (2017). Discovery of phylloseptins that defense against gram-positive bacteria and inhibit the proliferation of the non-small cell lung cancer cell line, from the skin secretions of Phyllomedusa frogs. Molecules.

[74] Zhu H., Ding X., Li W., Lu T., Ma C., Xi X. (2018). Discovery of two skin derived dermaseptins and design of a TAT-fusion analogue with broad-spectrum antimicrobial activity and low cytotoxicity on healthy cells. PeerJ.

[75] Agarwal S., Sharma G., Dang S., Gupta S., Gabrani R. (2016). Antimicrobial peptides as anti-infectives against *Staphylococcus epidermidis*. Med. Princ. Pract..

[76] Conlon J.M., Al-Ghaferi N., Abraham B., Leprince J. (2007). Strategies for transformation of naturally-occurring amphibian antimicrobial peptides into therapeutically valuable anti-infective agents. Methods.

[77] Giacometti A., Cirioni O., Ghiselli R. (2000). Polycationic peptides as prophylactic agents against methicillin-susceptible or methicillin-resistant *Staphylococcus epidermidis* vascular graft infection, Antimicrob. Agents. Chemother..

[78] Santajit S., Indrawattana N. (2016). Mechanisms of antimicrobial resistance in ESKAPE pathogens. BioMed Res. Int..

[79] O'Shea P. (2003). Intermolecular interactions with/within cell membranes and the trinity of membrane potentials: kinetics and imaging. Biochem. Soc. Trans..

[80] Jenssen H., Hamill P., Hancock R.E. (2006). Peptide antimicrobial agents. Clin. Microbiol. Rev..

[81] Lin B., Hung A., Li R., Barlow A., Singleton W., Matthyssen T., Sani M.A., Hossain M.A., Wade J.D., O'Brien-Simpson N.M., Li W. (2022). Systematic comparison of activity and mechanism of antimicrobial peptides against nosocomial pathogens. Eur. J. Med. Chem..

[82] Jiang Z., Vasil A.I., Vasil M.L., Hodges R.S. (2014). “Specificity Determinants” improve therapeutic indices of two antimicrobial peptides piscidin 1 and dermaseptin S4 against the gram-negative pathogens *Acinetobacter baumannii* and *Pseudomonas aeruginosa*. Pharmaceuticals.

[83] Pouny Y., Rapaport D., Mor A., Nicolas P., Shai Y. (1992). Interaction of antimicrobial dermaseptin and its fluorescently labeled analogues with phospholipid membranes. Biochemistry.

[84] Mukhopadhyay S., Bharath Prasad A.S., Mehta C.H., Nayak U.Y. (2020). Antimicrobial peptide polymers: no escape to ESKAPE pathogens-a review, world. J. Microbiol. Biotechnol..

[85] Wu Y., Yang N., Mao R., Hao Y., Teng D., Wang J. (2022). *In Vitro* pharmacodynamics and bactericidal mechanism of fungal defensin-derived peptides NZX and P2 against *Streptococcus agalactiae*. Microorganisms.

[86] Raheem N., Straus S.K. (2019). Mechanisms of action for antimicrobial peptides with antibacterial and antibiofilm functions. Front. Microbiol..

[87] Ong T.H., Chitra E., Ramamurthy S., Ling C.C.S., Ambu S.P., Davamani F. (2019). Cationic chitosan-propolis nanoparticles alter the zeta potential of S. epidermidis, inhibit biofilm formation by modulating gene expression and exhibit synergism with antibiotics. PLoS One.

[88] Pintavirooj C., Vongmanee N., Sukjee W., Sangma C., Visitsattapongse S. (2022). Biosensors for *Klebsiella pneumoniae* with Molecularly Imprinted Polymer (MIP) technique. Sensors.

[89] Malanovic N., Lohner K. (2016). Antimicrobial peptides targeting gram-positive bacteria. Pharmaceuticals.

